# Sodium Retention and Distribution in Growing and Adult Rodents Fed High and Low Salt Diets

**DOI:** 10.3390/nu18081212

**Published:** 2026-04-11

**Authors:** Christina Vialva, Sisi Cao, Song Yue, Linda H. Nie, Cheryl A. M. Anderson, Connie M. Weaver

**Affiliations:** 1 School of Exercise and Nutritional Sciences, San Diego State University, San Diego, CA 92182, USA; cvialva9227@sdsu.edu (C.V.);; 2 School of Health Sciences, Purdue University, West Lafayette, IN 47907, USA; yue42@purdue.edu (S.Y.); hnie@purdue.edu (L.H.N.); 3 Herbet Wetheim School of Public Health and Human Longevity Science, University of California San Diego, La Jolla, CA 92093, USA; c1anderson@health.ucsd.edu

**Keywords:** dietary salt, sodium, rat, mouse, sodium balance, sodium retention, Na-22, NAA

## Abstract

**Background/Objectives**: Previous research demonstrates higher sodium retention with increasing levels of dietary salt in some populations. Our objective was to determine whole-body sodium retention and sodium distribution on high and low salt diets using rodent models. **Methods**: Whole body retention of orally dosed Na-22, a gamma emitter, was measured in female growing and adult Sprague-Dawley rats on high (3.1% by wt. of diet) and low salt (0.13% by wt. of diet) diets. In a second study, whole-body sodium retention was compared between destructive inductively coupled plasma optical emission spectroscopy (ICP-OES) and neutron activation analysis (NAA) in adult male and female C57BL/6 mice. **Results**: Whole body retention of Na-22 was not different due to the age of rats on a high salt diet, but rats fed the high salt diet excreted Na-22 much more rapidly than rats fed a low salt diet. In mice, neither sodium retention nor tissue distribution was affected by dietary salt. Bland–Altman analysis indicated overall agreement between NAA and ICP-OES measurements, with observed systematic positive bias. **Conclusions**: Dietary salt had little effect on retention in normotensive rodents and should be studied in hypertensive models.

## 1. Introduction

Sodium reduction is a leading strategy to lower blood pressure and prevent hypertension [1,2,3]. Dietary sodium also negatively affects bone [4]. In part, these negative effects occur through the strong relationship between dietary sodium and urinary calcium excretion [5,6,7,8,9]. Avoiding negative calcium balance is important for reducing the risk of both hypertension and osteoporosis [10,11,12]. A controlled feeding study in adolescents on high and low sodium diets showed greater sodium retention and lower calcium retention on high salt diets in Black girls compared to White girls [2]. Because the Black girls did not gain weight or experience elevated blood pressure, the authors speculated that the Black growing adolescent girls sequestered sodium in bones along with minerals essential to growing bone. This sodium would be unavailable for water retention which otherwise could result in elevated blood pressure. Sequestering sodium into bone may be restricted to life stages of growth.

Investigating sodium homeostasis and tissue distribution at different life stages may give insights into vulnerability to hypertension [13]. Heer et al. [14] proposed that sodium could be sequestered in skin. In animal models, significant amounts of sodium can be sequestered in muscle, skin [3], and bone [15,16,17,18]. Rodent studies allow tissue-specific destructive analysis to test sodium accumulation in various anatomical structures, which are limited to end-of-sacrifice measurements. We have developed an In vivo Neutron Activation Analysis (IVNAA) technique for studying the in vivo distribution of sodium in animal models and humans [19,20,21]. IVNAA was recognized as a promising technique for quantifying whole body essential elements in the 1980s [22,23]; however, the technique has only more recently been applied to sodium [24].

We conducted two studies to (1) determine sodium retention and distribution in rodent models under high and low dietary salt interventions and (2) validate Neutron Activation Analysis (NAA) as a suitable method of sodium deposition by comparison with destructive chemical analysis by Inductively Coupled Plasma Optical Emission Spectroscopy (ICP-OES). In study 1, we tested the hypotheses that high salt intakes would lead to increased sodium retention in growing, but perhaps not mature, rodents. In study 2, we tested the hypotheses that sodium accumulation would be greater in muscle than in bone and skin in mature mice fed high salt diets compared to low salt diets and that NAA is a useful method for determining tissue sodium concentrations. A secondary aim was to test the hypothesis that normotensive rodents could be used as a model to determine the mechanisms that influence dietary sodium on blood pressure.

## 2. Materials and Methods

### 2.1. Na-22 Gamma Retention in Adult and Growing Rats Fed High and Low Salt Diets

A total of 39 female Sprague-Dawley (SD) rats (Harlan—Indianapolis, IN, USA) were individually housed in a controlled laboratory environment at Purdue University (West Lafayette, IN, USA). Half of the rats were skeletally mature (nine weeks of age and 200–225 g body weight) and half approximated puberty (five weeks of age and 75–99 g body weight) to mimic the adolescent girls discussed above [25]. After a one-week adaptation, rats were assigned to one of four groups: adult rats receiving a high sodium diet, adult rats receiving a low sodium diet, adolescent rats receiving a high sodium diet, and adolescent rats receiving a low sodium diet. A standard AIN-76 diet mixed with Modified AIN-76 Mineral Mix without sodium was modified by adding sodium chloride to achieve the “low salt” diet, 0.13% sodium (*w*/*w*), or the “high salt” diet, 3.10% sodium (*w*/*w*). The diet and water intake were ad libitum.

On day one, rats were given 0.1 µ Ci of Na-22, a gamma emitter, in water by oral gavage to track the whole-body retention of sodium over 23 days in response to its assigned diet. The residual Na-22 tracer in individual rats was measured in a full-body counter within four hours after gavage and every twelve hours, post-gavage, as previously described [26]. Na-22 was counted using a photopeak of 900–1700 keV.

### 2.2. Measuring Tissue-Specific Sodium in Adult C57BL/6 Mice Fed Low and High Salt Diets

In total 24 adult (8 weeks old) male and female C57BL/6 mice (Charles River—Hollister, CA, USA) were housed at San Diego State University (San Diego, CA, USA) as same-sex pairs in a controlled laboratory setting before half were randomized by body weight to either a high (3.1% *w*/*w*) or low (0.13% *w*/*w*)-salt diet for two weeks. Consumption of diet and water intake were ad libitum. Diet intake was measured every two days for each pair, and individual consumption was estimated as the mean intake of the pair.

After sacrifice, using the method of controlled carbon dioxide asphyxiation, the mice were stored face-down in polyethylene capped vials at −20 °C, before being subjected to Neutron Activation Analysis (NAA) to study whole-body sodium distribution.

NAA measurements were performed on 5 male mice fed the low salt diet and 5 male mice fed the high salt diet. The mice were harvested for the following tissues: right paws, skin from the abdomen, right femurs, quadricep muscle, and L1–L5 vertebrae. The residual carcass was also used to approximate whole-body sodium as a comparison against the results of the NAA. Each tissue was then ashed in a muffle furnace at 600 °C for a minimum of three days, before being dissolved in trace mineral grade concentrated nitric acid and diluted to a final 5% concentration to be measured on an Agilent 5800 Inductively Coupled Plasma Optical Emission Spectrometer (ICP-OES, Agilent Technologies—Santa Clara, CA, USA) for sodium.

### 2.3. Measuring Whole-Body Sodium with NAA

Five low salt and five high salt-fed male mice were selected to compare the measurements of whole-body sodium in the mice between ICP-OES and NAA.

The NAA sodium measurement system consisted of a deuterium-deuterium (DD) neutron generator (Adelphi Technology Inc., Redwood City, CA, USA) based irradiation component and a high-efficiency HPGe detector (AMETEK, Berwyn, PA, USA). The DD109M neutron generator produced 2.45 MeV neutrons that passed through a beam shaping assembly that consisted of a moderator, reflector, and shield. The purpose of this assembly was to thermalize the neutrons, reflect maximum thermal neutron flux to the site of irradiation, and reduce the dose inside the irradiation cave (for in vivo measurement) and outside the shielding to an acceptable level.

Each carcass, oriented face-down in a polyethylene vial, was positioned inside the customized assembly, where the thermal neutron flux was optimized. Irradiation occurred for 20 min, followed by a 5 min-long decay period, and an additional hour for counting.

To calibrate the NAA, a standard curve of saline phantoms was prepared, ranging from 0 to 400 PPM Na.

### 2.4. Sample Size Determinations and Data Handling

This study was exploratory for determining power calculations for future studies, as effect sizes were unknown. The sample size for Study 1 was based on sample sizes used in previous studies for whole body retention of Zn-65 and Fe-59 in rats with *n* = 6 to 10 per group, lacking previous data using Na-22 [27,28]. The sample size for Study 2 was based on a study of NAA in humans, which studied 7 subjects [20].

Microsoft Excel (Office 365) and JASP (Version 0.96) were used to perform statistical analyses, with alpha (α) = 0.05 being considered statistically significant for all tests. Results are reported as mean ± standard deviation (SD), unless otherwise noted. Holm post hoc tests were used for every ANOVA performed.

In the rat study, a three-way repeated measures ANOVA with factors of age, salt level, and time was also performed to check if there were any statistical differences in sodium retention among groups in the rat study.

In study 2, a Bland–Altman graph was prepared to compare total whole-body sodium group means in mice measured by ICP-OES and NAA. A two-way ANOVA with factors of sex and salt level checked for differences in total whole-body sodium. A three-way repeated measures ANOVA with factors of salt level, sex, and time measured for any differences in body weight among the groups. Linear regression was used to test for an association between cumulative sodium intake and total sodium in the carcass for both sexes. Two-way repeated measures ANOVA with factors of salt level and sex was performed to check if there were any differences in tissue-specific sodium concentration.

## 3. Results

### 3.1. Study 1: Whole-Body Sodium Retention in Adolescent and Adult Rats

**Figure 1:**
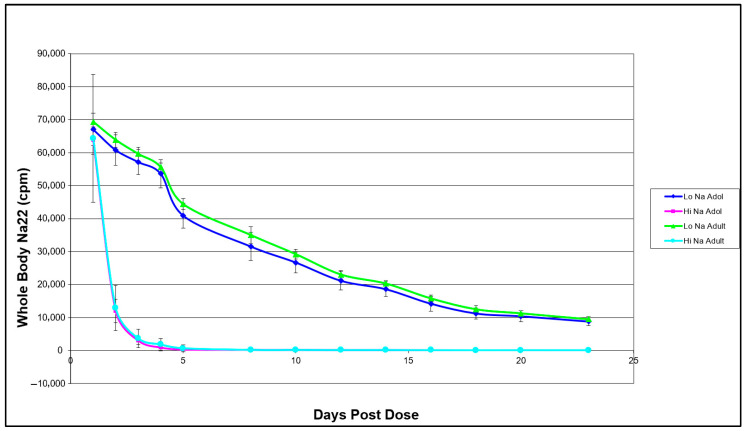
Whole-body Na-22 retention as affected by age and sodium intake in female Sprague–Dawley rats. Points represent mean ± SD (*n* = 10 per group, except the adult high-salt group, where *n* = 9). A three-way repeated measures ANOVA with factors of age, salt level, and time resulted in a significant salt level * time interaction. A Holm post hoc test of the interaction of salt level * time showed that rats fed with high salt and low salt diets had different whole-body Na-22 retention after day 1, regardless of age (*p* < 0.001).

Whole-body Na-22 retention over the 24-day study period was directly influenced by dietary sodium intake but not by developmental stage. Figure 1 illustrates the decay of Na-22 gamma emissions as a function of time across the four experimental groups. The three-way repeated measures ANOVA with factors of age, salt level, and time resulted in a significant salt level * time interaction. A Holm post hoc test of the interaction of salt level * time showed that rats fed with high salt and low salt diets had different whole-body Na-22 retention after day 1.

### 3.2. Tissue-Specific Sodium Deposition in Adult Mice

**Figure 2:**
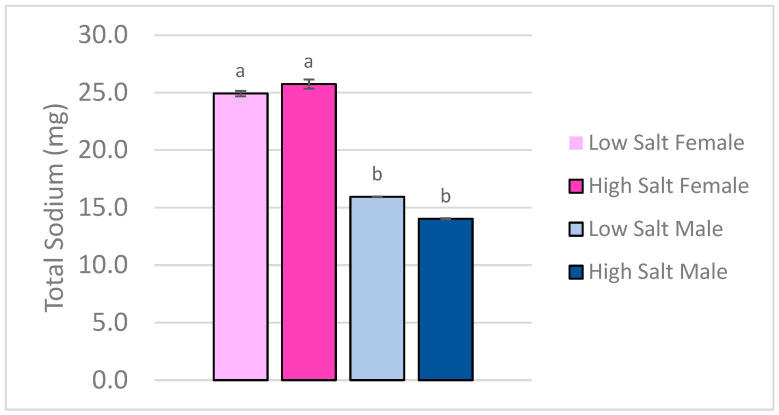
Total Sodium in Carcasses of Adult C57/BL6 Mice measured by ICP-OES. Bars represent mean values; error bars indicate SD (*n* = 5/group). Bars with different letters indicate statistically significant differences between groups (*p* < 0.05) by two-way ANOVA with factors of salt level and sex.

Total carcass sodium content, as determined by ICP-OES, was not different between the high and low salt diet groups (*p* > 0.05), suggesting that short-term dietary sodium loading did not alter overall body sodium levels in mature mice. However, there was a significant main effect of sex (*p* < 0.05), with female mice retaining more total sodium than male mice, regardless of assigned diet (Figure 2). Furthermore, there was no significant association between cumulative sodium intake and total sodium in the carcass (*p* = 0.563). Carcass sodium differed significantly by sex (*p* < 0.001), with females exhibiting higher levels than males, independent of diet. Salt level was not a significant predictor (*p* = 0.640).

**Figure 3:**
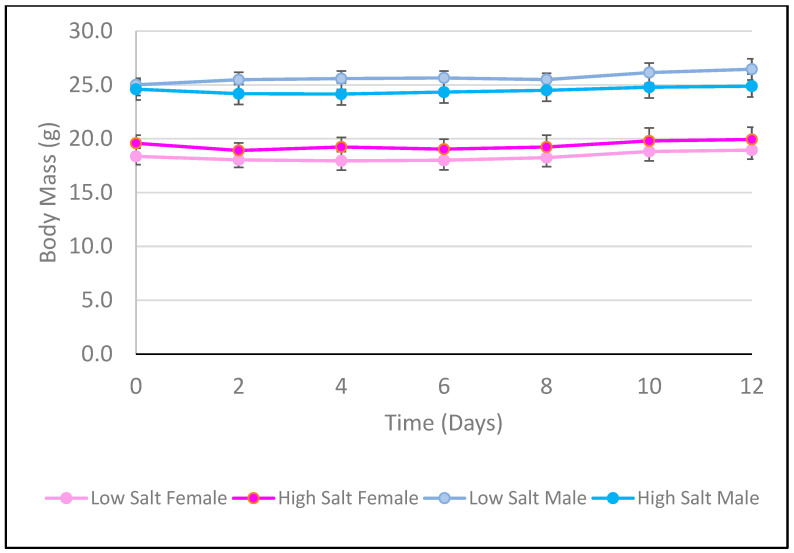
Average Body Weight of C57BL/6 Mice per Feeding Group over Study Duration. Points represent mean values; error bars indicate SD (*n* = 6/group). A three-way repeated measures ANOVA with factors of time, sex, and salt level measured for differences in body weight over the study duration. There was no difference in body weight within sex, regardless of salt level (*p* > 0.05).

**Figure 4:**
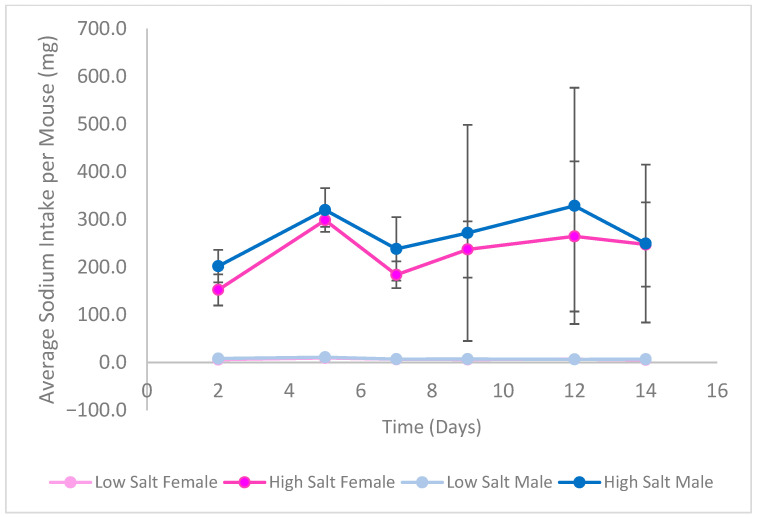
Average sodium intake of C57BL/6 Mice per Feeding Group over Study Duration. Points represent mean values; error bars indicate SD (*n* = 6/group). A three-way repeated measures ANOVA with factors of time, sex, and salt level measured for differences in sodium intake over the study duration. Mice fed the high salt diet consistently consumed more sodium than those fed the low salt diet, regardless of sex (*p* < 0.05).

Figure 3 and Figure 4 show the average weight gain and sodium intake for each group of mice over the study duration, respectively. Neither female nor male mice differed in their weight change throughout the study (*p* > 0.05). The mice on the high salt diet consistently consumed more sodium than those on the low salt diet, regardless of sex (*p* < 0.05).

**Figure 5:**
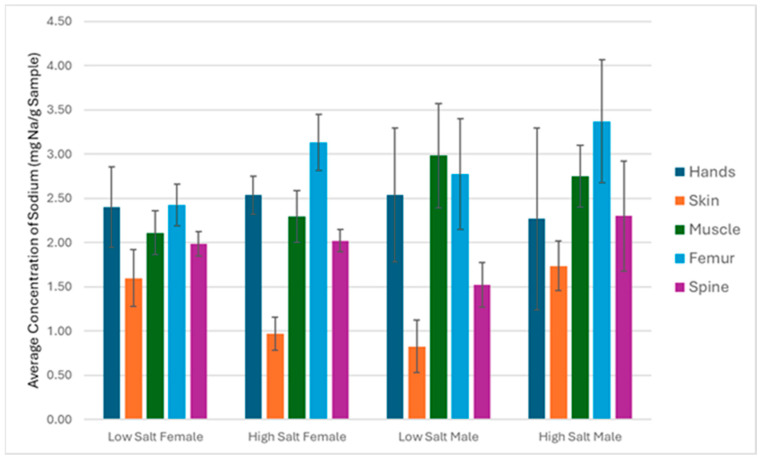
Concentration of sodium in isolated tissues of adult C57BL/6 mice measured by ICP-OES on a wet weight basis. Bars represent mean ± SD (*n* = 5 per subgroup, except male skin and muscle in the low- and high-salt groups, where *n* = 4 per tissue). Two-way repeated measures ANOVA with factors of salt level and sex was performed to check if there were any differences in tissue-specific sodium concentration. No differences among tissues were found (*p* > 0.05).

Neither dietary salt nor sex impacted individual tissue sodium concentration (*p* > 0.05) (Figure 5). Skin had the lowest sodium concentration of the tissues analyzed.

### 3.3. Validation of NAA with ICP-OES

**Figure 6:**
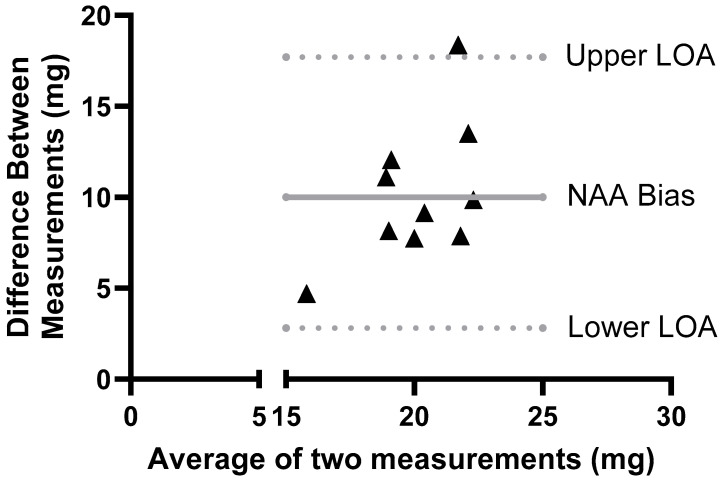
Bland–Altman Comparison of Total Sodium in Adult Male Mice (N = 10) Fed High and Low Salt Diets Measured by ICP-OES and NAA (Mean Difference = 10.25 ± 3.8 mg Na).

A comparison of whole-body sodium estimates obtained via NAA and ICP-OES using a Bland–Altman comparison shows that most differences between the methods fell within the 95% limits of agreement. (Figure 6). As summarized in Figure 6 and Table 1, the largely positive bias between NAA and ICP-OES methods shows that NAA systematically measures higher total sodium content than the ICP-OES, with a mean bias of 10.25 mg Na.

## 4. Discussion

In two rodent studies, developmental stage and sex-specific differences in sodium retention and tissue distribution under controlled high and low sodium diets were evaluated. We also validated the performance of Neutron Activation Analysis (NAA) as a non-destructive method for assessing whole-body sodium.

Contrary to our first hypothesis (Study 1), we found little evidence that adolescent rodents retain more sodium than adults when exposed to high dietary sodium over a two-week period. This contrasts with prior human data suggesting that growing adolescents, particularly Black girls, may sequester sodium in bone without corresponding increases in blood pressure or weight gain [25,29]. The discrepancy with the human study cannot be explained by a difference in the intervention period. The intervention periods of the human and rat study were nearly identical at 21 days for the human study and 23 days for the rat study. In humans, sodium and water homeostatic regulation of an acute saline infusion takes approximately two days [30]. Linear growth is different in rodents than in humans in that rodents do not have a sharply defined peak growth period at puberty and continue to grow as adults. Nevertheless, the ages we selected in our rat study correspond to rapid and slow growth periods. Our findings also contrast with a previous report that older age correlates with greater sodium storage in skin by Na-23 resonance imaging of peritoneal and hemodialysis patients [31,32].

In agreement with Study 1, adult mice (Study 2) did not have higher total body or more tissue sodium levels in response to high salt diets. Thus, our second hypothesis—that sodium loading would increase sodium accumulation in muscle more than in bone or skin—was not supported. This contrasts with earlier findings that suggested preferential sodium storage in muscle under high sodium conditions^3^, as well as with Crescenzi et al., who found no correlation between dietary sodium and muscle or skin sodium content in patients (with lipedema) [33]. Collectively, these results reinforce the idea of tight physiological regulation of sodium distribution under non-pathological conditions.

Significant sex differences were also observed, such that female mice had higher total body sodium than males. This finding highlights the influence of body composition and potentially sex hormones on sodium homeostasis. Sex effects on sodium handling have been reported by Selvarajah et al., in which human women experienced greater sodium to potassium ratios in the skin compared to men after one week of slow-paced salt loading by consumption of salt tablets [34]. Furthermore, the effect of hormones on sodium regulation has been thoroughly explored, notably through ovariectomized (OVX) rat models that simulate a post-menopausal condition. A long-term (6 weeks) estradiol treatment on OVX Sprague-Dawley resulted in suppressed renal epithelial sodium channel (ENaCα) expression, reducing blood pressure, while also finding that aldosterone levels recovered from what had been reduced from the OVX surgery [35].

The physiological basis for sodium regulation may relate to the “Natriuretic-Ureotic Principle” proposed by Minegishi et al., which describes a coordinated mechanism by which sodium is excreted via both natriuresis and enhanced urea production [36]. This principle links hepatic urea cycle activation to renal sodium handling in a “parallel movement” of water into the urine, coupled with the renal concentration process, which has antiparallel water reabsorption. Particularly under high-salt conditions, rodents may reflect compensatory hepatic ureagenesis, enabling sodium excretion without significantly altering total body sodium or tissue-level stores. This stimulation likely explains the more rapid excretion of Na-22 we observed under high salt feeding conditions.

NAA-derived measurements showed concordance with ICP-OES, although with a large bias in Bland–Altman plots, with a systematically higher value for sodium concentration by NAA of 10.25 on average compared to ICP-OES values. The correction value in our study should be interpreted with caution because of the narrow range in carcass sodium content and small sample size. A larger sample size over a wider range of sodium concentrations that might only be achievable in states of disordered sodium handling is needed to confirm this.

This study has several limitations. First, NAA as used in our study does not resolve compartment-specific sodium distribution (e.g., bone vs. muscle), necessitating destructive techniques like ICP-OES for tissue-level analysis, or approximation through techniques such as Na-23 resonance imaging. Unlike for mice, larger animals or humans would result in two decay curves thought to represent sodium in bone vs. soft tissue^6^. The range of sodium concentrations in the carcasses of our study may have been too narrow to adequately validate NAA. Also, only male mice were measured with NAA. Second, all sodium values reported through ICP-OES measurements were based on wet tissue weight, which is subject to variability due to hydration status during weighing. Third, food intake for the mouse study was measured within caged pairs rather than individually. Therefore, individual cumulative sodium intake was estimated as the mean intake per pair, assuming equal consumption between mice. This may introduce variability in individual intake estimates and should be considered when interpreting the results of the linear regression. Also, small subgroup sizes, especially in sex-stratified analyses, have limited power to detect more nuanced effects. However, the sample sizes reported here are comparable to other studies of this type, and there was no trend to indicate that an increased sample size would reveal a dietary sodium effect on sodium tissue retention or distribution. Finally, studying the effect of dietary salt on tissue sodium levels may have impacts in conditions of disordered sodium handling, such as salt-sensitive rodent strains that were not observed in normotensive, healthy rodents.

## 5. Conclusions

Dietary sodium had little impact on sodium retention or tissue distribution in normotensive rodent models. This may explain the failure of salt intervention studies to find an association with blood pressure in healthy populations [29,31,37,38]. The female sex, older age, and hypertension all increase the sensitivity to dietary sodium interventions [38]. This study also challenges the notion that sodium retention is developmentally elevated during adolescence—at least in rodents—and highlights the physiological robustness of sodium homeostasis across moderate dietary conditions and growth stages. Short-term studies such as the one reported here can provide insight into physiological changes that result from changes in diet. But longer-term studies are required to fully understand the relevance to hypertension and other health conditions.

Although promising, further research to validate the use of NAA for small animal models with application in humans is needed under conditions that produce a wider range in sodium tissue distribution. In particular, salt-sensitive rodent models or older patients with hypertension would provide insights into the robustness of NAA to determine sodium distribution in vulnerable populations where sodium homeostasis is dysfunctional.

## Figures and Tables

**Table 1:** Whole-body Total Sodium in Male C57BL/6 Mice Measured by ICP-OES and NAA (N = 10/measurement).

Parameter	ICP-OES Mean Total Sodium (SD)	NAA Mean Total Sodium (SD)	Mean Difference (SD)	95% Limits of Agreement
Whole-body Total Sodium (mg)	15.0 (1.9)	25.2 (3.4)	−10.25 (3.8)	−17.7, −2.8

## Data Availability

The raw data supporting the conclusions of this article will be made available by the authors on request.

## Abbreviations

The following abbreviations are used in this manuscript:

NAA	Neutron Activation Analysis
ICP-OES	Inductively Coupled Plasma Optical Emission Spectroscopy
OVX	Ovariectomized
SD	Standard Deviation
ENaCα	Alpha subunit of the epithelial sodium channel
